# Novel Caffeine-Assisted Aqueous Extraction Strategy for Enhanced Solubilization of Polycyclic Aromatic Hydrocarbons from Creosote-Treated Waste Wood

**DOI:** 10.3390/molecules31173107

**Published:** 2026-09-04

**Authors:** Željka M. Nikolić, Nebojša R. Radović, Aleksandra M. Radulović, Milica P. Marčeta Kaninski, Vladimir M. Nikolić, Dragana Z. Živojinović

**Affiliations:** 1 Institute of General and Physical Chemistry, Studentski trg 12/V, 11000 Belgrade, Serbia; aradulovic@iofh.bg.ac.rs (A.M.R.); milica@iofh.bg.ac.rs (M.P.M.K.); vnikolic@iofh.bg.ac.rs (V.M.N.); 2 Faculty of Chemistry, University of Belgrade, Studentski trg 12-16, 11000 Belgrade, Serbia; nradovic@chem.bg.ac.rs; 3 Faculty of Technology and Metallurgy, University of Belgrade, Karnegijeva 4, 11000 Belgrade, Serbia; gaga@tmf.bg.ac.rs

**Keywords:** railroad ties, solvent extraction, caffeine, polycyclic aromatic hydrocarbons

## Abstract

This study evaluates caffeine-assisted aqueous extraction systems for the enhanced extraction of Σ16 US EPA priority polycyclic aromatic hydrocarbons (PAHs) from waste creosote-infused wood. Aqueous solutions of eight water-soluble organic acids were investigated with and without caffeine to assess its effect on PAH extraction. Caffeine improved extraction across all systems, but the extent of this improvement varied depending on the initial performance of each extraction agent without caffeine. Deionized water exhibited a negligible extraction yield (0.0027%), whereas caffeine addition increased the yield to 1.6886%, corresponding to a 625-fold improvement. Despite substantial differences among agents without caffeine, most caffeine-containing systems achieved similar extraction yields (4.2–5.3%), indicating that caffeine-induced hydrotropic solubilization became the dominant factor governing PAH extraction. Based on the maximum extraction performance achieved in the presence of caffeine, the investigated systems can be ranked as follows: acetic acid-caffeine > citric acid-caffeine > p-TsOH-caffeine ≈ tartaric acid-caffeine ≈ lactic acid-caffeine ≈ formic acid-caffeine > ascorbic acid-caffeine. These findings identify caffeine-assisted aqueous extraction as a promising approach for enhancing PAH extraction from creosote-treated waste wood while reducing reliance on conventional organic solvents in the primary extraction step.

## 1. Introduction

Creosote-treated waste wood represents one of the most challenging hazardous wood waste streams due to the persistence and toxicity of creosote-derived polycyclic aromatic hydrocarbons (PAHs). Wood has long been used as a construction material in railway infrastructure due to its favorable mechanical properties, availability, and cost-effectiveness. To extend its service life under severe environmental conditions, wooden railroad ties have traditionally been pressure-impregnated with coal tar creosote, a complex mixture composed predominantly of PAHs, together with phenolic and heterocyclic compounds [1,2]. Creosote provides excellent protection against fungal decay, insects, and weathering, enabling service lifetimes of several decades. However, the persistence and toxicity of its constituents have raised increasing environmental and human health concerns. Many PAHs are persistent and are associated with carcinogenic, mutagenic, reproductive, and immunotoxic effects. Among the 16 US EPA priority PAHs, compounds such as benzo[a]pyrene, benzo[a]anthracene, and chrysene have been specifically associated with adverse health effects [3,4]. Large quantities of creosote-treated railroad ties reach the end of their service life annually, generating significant amounts of hazardous wood waste, such as waste railroad ties (WRTs). The presence of creosote-derived contaminants, particularly PAHs, restricts their direct reuse and recycling. Therefore, the development of sustainable treatment strategies for contaminant removal is essential to enable safe valorization of this waste stream and promote circular economy approaches [5,6].

Within the European Union, the production, marketing, and use of creosote and creosote-treated wood are subject to strict regulatory control. Under the REACH framework, creosote is restricted to specific industrial and professional applications, while its use in consumer products and in applications involving frequent human contact is prohibited [7]. Creosote-treated wood is therefore permitted only for limited applications, such as railway infrastructure, utility poles, agricultural fencing, and certain marine structures, with additional restrictions on reuse and secondary use [8]. Upon reaching the end of their service life, creosote-treated railroad ties enter a waste management stream and are regulated within the framework of the U.S. EPA Non-Hazardous Secondary Materials (NHSM) rule (2011), including subsequent amendments in 2013, 2016, and 2018 [9]. Although these materials retain potential value for energy or material recovery, their management and reuse are constrained by the presence of creosote-derived contaminants, mostly PAHs, which may pose environmental and health risks if not appropriately controlled [10]. PAHs are a major group of persistent organic contaminants present in coal tar creosote and are considered the primary environmental concern associated with creosote-treated wood [11,12,13]. These compounds consist of fused aromatic rings and exhibit a wide range of physicochemical properties depending on their molecular structure. Low-molecular-weight PAHs containing two to three aromatic rings generally show higher volatility and water solubility (with values ranging from approximately 0.045 to 31 mg/L), whereas high-molecular-weight PAHs are characterized by greater hydrophobicity, stronger sorption behavior, and increased environmental persistence with water solubility values ranging from approximately 0.00019 to 0.26 mg/L [14,15].

The environmental behavior of PAHs is largely governed by their hydrophobic nature and strong affinity for organic matrices. In creosote-treated wood, PAHs are retained within the lignocellulosic structure through interactions with wood polymers and hydrophobic domains, resulting in slow release and long-term persistence. During the service life of treated wood and after disposal, PAHs may migrate from the wood matrix into surrounding soil, sediment, and water, posing potential risks to terrestrial and aquatic ecosystems [16,17,18]. Their low biodegradability, environmental persistence, and adverse toxicological properties make PAHs contaminants of major environmental concern [19,20]. Consequently, the 16 polycyclic aromatic hydrocarbons designated as priority pollutants by the US EPA are commonly used as target analytes for evaluating the contamination of environmental matrices and the efficiency of remediation technologies [21]. Several treatment and management strategies have been proposed for WRTs, including thermal treatment, solvent extraction, material recycling, reuse and energy recovery. Thermochemical processes, such as pyrolysis [22,23], torrefaction [24], gasification [25,26] and thermal desorption [27], have shown considerable potential for recovering creosote components from WRTs [28,29]. However, these technologies generally require high operating temperatures and high energy consumption, often resulting in irreversible degradation of the wood matrix and limiting opportunities for subsequent material reuse [30].

Among the available separation techniques, solvent extraction is widely employed for the isolation of valuable compounds from natural materials, purification processes, and sample preparation for instrumental analysis [31,32]. Solvent extraction has been investigated in our previous work [33] as a promising approach for the selective removal of organic contaminants while largely preserving the structural integrity of the wood. Conventional extraction procedures predominantly employ organic solvents, such as dichloromethane, acetone, hexane, and toluene, owing to their high affinity for hydrophobic PAHs and consequently high extraction performances [34,35]. However, the use of these solvents is associated with significant environmental, health, and safety concerns, including toxicity, volatility and flammability. These drawbacks motivate the development of aqueous extraction systems capable of enhancing the apparent aqueous solubility of PAHs while reducing reliance on hazardous organic solvents in the primary extraction step. Such systems are relevant to green chemistry [36,37,38] efforts to reduce hazardous solvent use, although their overall environmental performance requires process-to-level assessment.

Water is an attractive extraction medium due to its low cost, non-toxicity, non-flammability, ease of handling and environmental compatibility [39]. However, the limited solubility of hydrophobic pollutants in water often restricts extraction performance. Its practical application for PAH extraction is severely limited by the extremely low aqueous solubility of these hydrophobic compounds [40]. Hydrotropic agents have been investigated as aqueous solubilization agents and as alternatives to conventional organic solvents.

Hydrotropic agents, including various organic acid salts, function by disrupting the structured network of water and promoting favorable interactions with target analytes, thereby increasing their apparent aqueous solubility [41,42,43,44,45]. Hydrotropy represents an attractive strategy for increasing the apparent aqueous solubility of poorly water-soluble compounds through the addition of highly water-soluble amphiphilic molecules [46]. Unlike surfactants, hydrotropes generally do not form micelles at low concentrations but enhance solubilization through weak, cooperative intermolecular interactions, including π-π stacking, hydrogen bonding, and self-association [47,48]. Hydrotropes organize into less ordered, dynamic structures at relatively high concentrations. This mechanism enables their application in pharmaceuticals, chemical industries, extraction processes, and green chemistry, where they are used to improve solubility and process efficiency without relying on conventional surfactants [49]. Among naturally occurring hydrotropes, caffeine has attracted increasing attention because of its ability to enhance the apparent solubility of aromatic and hydrophobic compounds [50,51]. The hydrotropic effect of caffeine originates primarily from π-π interactions between its conjugated aromatic system and aromatic solutes, accompanied by self-association of caffeine molecules in aqueous solution [52]. Hydrotropic solutions have been successfully employed to improve the aqueous solubility of various pharmaceutical compounds, dyes, and PAHs in analytical and pharmaceutical applications [51,53,54]. The incorporation of caffeine as a hydrotropic agent offers a means of increasing the apparent aqueous solubility of hydrophobic PAHs while retaining water as the primary extraction medium. In combination with selected biodegradable organic acids, this provides a basis for evaluating such systems as aqueous alternatives to conventional solvent extraction. These material characteristics are relevant to selected green chemistry considerations but do not, by themselves, establish the overall sustainability of the process [38].

The selection of the investigated organic acids was guided by the physicochemical properties, anticipated extraction performance, and reported attributes relevant to solvent selection. Eight acids, formic, acetic, citric, lactic, tartaric, ascorbic, oxalic, and p-toluenesulfonic acid, were chosen to represent a broad range of acid strengths, molecular structures, and environmental characteristics [55,56,57]. Simple monocarboxylic acids were included as conventional extraction agents, whereas naturally occurring multifunctional hydroxy acids were selected as promising green alternatives because of their low toxicity, high biodegradability, and, in most cases, production from renewable feedstocks, consistent with the principles of using safer solvents and renewable resources [38,58,59,60]. Oxalic acid was incorporated owing to its strong complexing ability, which may facilitate the mobilization of target compounds through interactions with the wood matrix [61,62]. In contrast, p-toluenesulfonic acid, a strong aromatic organic acid, was included as a chemically distinct reference system for benchmarking extraction performance [63].

To the best of our knowledge, the combined use of biodegradable organic acids and caffeine as an entirely aqueous hydrotropic extraction system for the removal of PAHs from creosote-treated railroad ties has not been previously reported. Therefore, the aim of this study was to develop and systematically evaluate novel water-based extraction media composed of organic acids and caffeine for the efficient extraction of Σ16 US EPA PAHs from waste creosote-treated railroad ties. The effects of the acid type, caffeine addition, extraction time, and multistage extraction were investigated to assess the performance of these systems as the primary extraction step.

## 2. Results and Discussion

The extraction performance of the investigated aqueous extraction systems was systematically evaluated using creosote-treated waste railway ties as the test material. Particular attention was given to the influence of the extraction medium composition, caffeine addition, extraction time, and multistage extraction on the extraction yield of Σ16 US EPA PAHs.

### 2.1. The First Set of Tests: Effect of Caffeine Addition on PAH Extraction Performances from Creosote-Treated Wood in Different Extraction Media

The first set of experiments involved the preparation of aqueous extraction agents based on deionized water, eight water-soluble organic acids, and caffeine. A comparative evaluation of the extraction performance of aqueous organic acid solutions, both in the absence and presence of caffeine, was conducted. The extraction yield of Σ16 US EPA PAHs was expressed as a percentage relative to the initial mass of the test sample. Several organic acid solutions were prepared at different concentrations, while the same amount of caffeine was added to the corresponding solutions whenever possible. In some cases, the amount of dissolved caffeine was limited by its solubility under the applied experimental conditions, including room temperature (25 °C) and the chemical nature of the organic acid medium.

For the preparation of the aqueous extraction solutions, eight water-soluble organic acids, deionized water, and caffeine were used as extraction agents. The organic acids selected for the preparation of the extraction solutions were concentrated formic acid (85%), concentrated acetic acid (99.9%), concentrated lactic acid (85%), citric acid, L-ascorbic acid, oxalic acid, tartaric acid, and p-toluenesulfonic acid. A series of extraction systems with different chemical compositions was investigated to assess the individual and combined effects of organic acids and caffeine on PAH extraction. The composition of each system, including the main extraction components and their concentrations, is presented in Table 1.

The influence of caffeine addition on the PAH extraction performances was evaluated by comparing the extraction yields obtained with aqueous extraction agents in the absence and presence of caffeine. The compositions of the S0–S13 extraction systems are given in Section 3.5.

The extraction yields of the Σ16 US EPA PAHs for all investigated systems, together with the corresponding increase after caffeine incorporation, are presented in Table 1.

The extracted yield of the sum of the 16 US EPA PAHs, EY, was calculated using the following Equation (1):

(1) $$EY\left(\%\right)=\frac{\Sigma PAHs\left(\frac{µg}{{cm}^{3}}\right)\times D_{f}\times V({cm}^{3})}{m_{s}(g)\times 10000}$$

where *ΣPAHs* is the total concentration of the sixteen US EPA PAHs, *D_f_* is the dilution factor, *V* is the extract volume in n-hexane and *m_s_* is the mass of the test sample.

**Figure 1:**
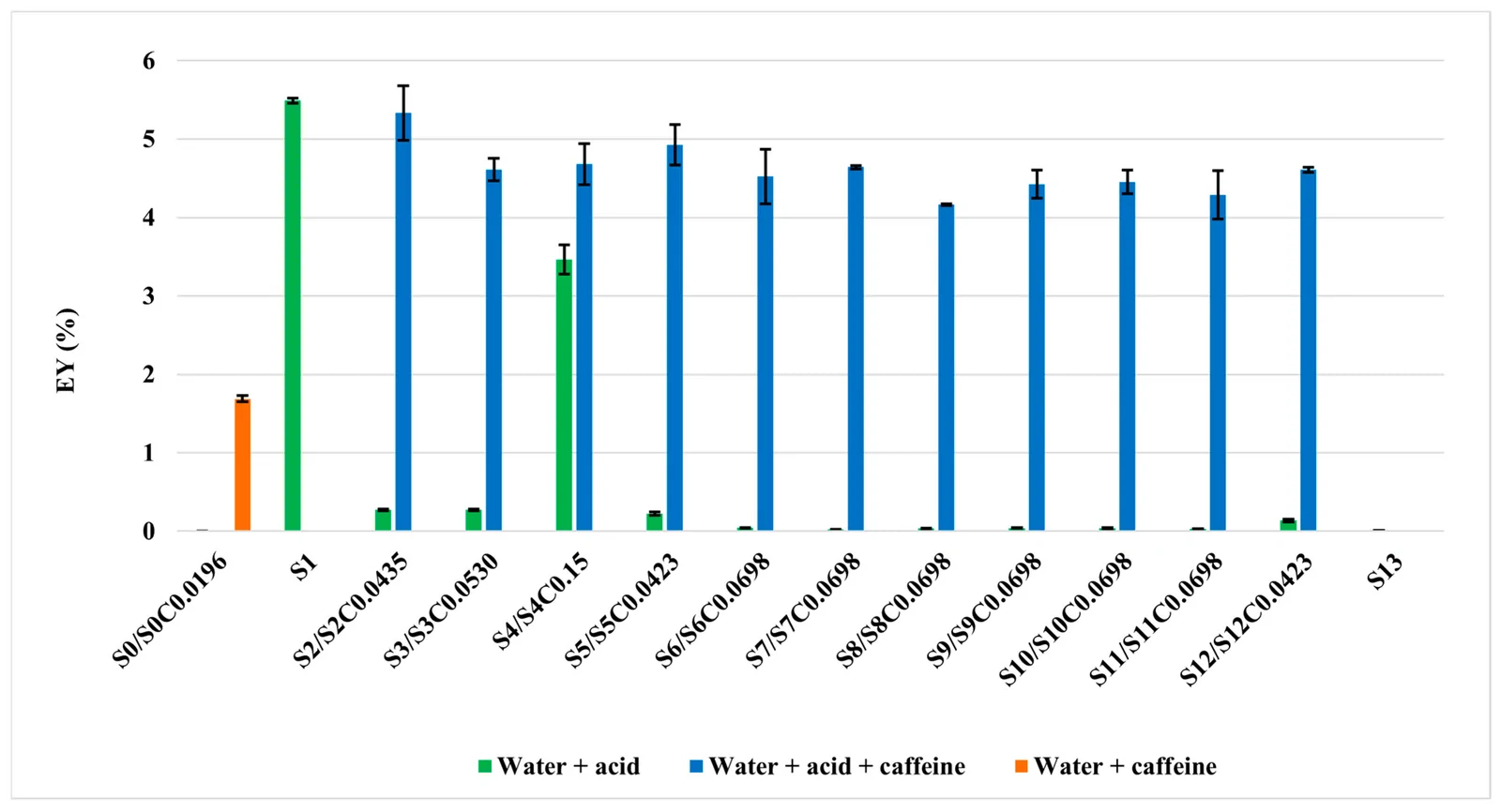
Comparison of the extraction yield of Σ16 US EPA PAHs obtained using aqueous extraction agents without and with caffeine. Error bars represent the standard deviation (SD) of two independent measurements (n = 2). The absence of error bars in some data indicates low standard deviation values.

Concentrated acetic acid (S1) and 7.5 wt% oxalic acid solution (S13) did not have a pair with caffeine because the caffeine does not dissolve in them. S1 and S13 were evaluated only in the absence of caffeine. The obtained results are presented in Figure 1 as a bar chart illustrating the EY values of the Σ16 US EPA PAHs obtained with the investigated aqueous extraction systems, allowing direct comparison of the extraction performance of caffeine-free and caffeine-containing media.

The obtained results demonstrate that caffeine is the dominant factor governing the extraction capability of PAHs in aqueous systems. In the absence of caffeine, most extraction media exhibited limited capacity for PAH solubilization, with EY values generally below 1%, indicating that acidic aqueous systems alone are insufficient for the effective extraction of hydrophobic PAHs. Following caffeine addition, the extraction yields increased substantially, reaching approximately 4–5.5% in most systems, confirming the strong hydrotropic effect of caffeine. Although the type and concentration of organic acid influenced the extraction performance, their effect was considerably less pronounced compared with the influence of caffeine. The convergence of extraction yields observed among caffeine-containing systems indicates that caffeine-mediated solubilization becomes the dominant mechanism controlling PAH transfer into the aqueous phase. This behavior is attributed to hydrotropic aggregation and non-covalent interactions, including possible π-π interactions between the aromatic structure of caffeine and PAH molecules, which increase the apparent solubility of otherwise poorly water-soluble compounds.

To quantify the contribution of caffeine to PAH extraction, an enhancement factor was calculated by taking the corresponding caffeine-free extraction system as the reference. The enhancement factor, EF, was defined as the ratio between the PAH extraction yield obtained with caffeine and that obtained for the corresponding decaffeinated system, according to Equation (2):

(2) $$EF=\frac{EY\;of\;caffeine\;containing\;solution(\%)}{EY\;of\;corresponding\;caffeine\;free\;solution(\%)}$$

This parameter was used to evaluate the relative improvement in extraction performance resulting from caffeine addition for each extraction system.

The S0 system (deionized water) exhibited a negligible extraction yield of 0.0027%, representing the intrinsic solubility limitation of PAHs in an aqueous medium. In contrast, the S0C system containing caffeine achieved an extraction yield of 1.6886%, corresponding to approximately a 625-fold increase compared with water alone. This result confirms that caffeine can act as an effective hydrotrope even in the absence of organic acids by enhancing PAH solubilization through interactions with caffeine and the formation of soluble molecular aggregates. The greatest relative improvement was observed for the S0/S0C pair. However, the extraction performance of S0C remained lower than that of the caffeine–organic acid systems. This difference can be attributed to the limited aqueous solubility of caffeine, resulting in a lower dissolved caffeine concentration compared with acidic media where caffeine solubility is enhanced. The extraction yields obtained with caffeine-free acidic systems varied considerably, ranging from 0.0121% (S13) to 5.4907% (S1). The low yield obtained for most organic acids confirms that acidity alone does not provide sufficient solubilization of hydrophobic PAHs. The higher extraction capability observed for S1 and S4 indicates that the chemical nature of the acid can influence PAH release; however, this effect is secondary compared with the contribution of caffeine.

The introduction of caffeine into organic acid-based extraction media resulted in a substantial increase in PAH extraction performance. Most caffeine-containing systems achieved yields between 4.17% and 5.33%, despite large differences among the corresponding caffeine-free systems. For example, systems with initially poor extraction performance, such as S6–S11, exhibited more than 100-fold increases after caffeine addition, whereas systems with already high extraction yields showed smaller relative improvements. The S4/S4C system represents the clearest example, where the extraction yield increased only 1.35-fold due to the relatively high capability of the caffeine-free medium. This trend demonstrates that caffeine primarily enhances systems with limited intrinsic extraction capability, compensating for the poor solubilization ability of the original aqueous media. Once a sufficient amount of dissolved caffeine is present, the extraction performance becomes largely independent of the specific organic acid used. The narrow range of extraction yields observed among caffeine-containing systems (approximately 4.2–5.3%) confirms that caffeine-mediated hydrotropic solubilization is the main factor controlling PAH extraction performance. Overall, these findings indicate that caffeine addition transforms chemically different aqueous extraction systems into comparable high-capability media, reducing the influence of individual organic acids and highlighting the dominant role of caffeine in PAH solubilization.

### 2.2. Second Set of Tests: Extraction Using Organic Acid-Caffeine Solutions of Different Caffeine Mass Fractions

**Figure 2:**
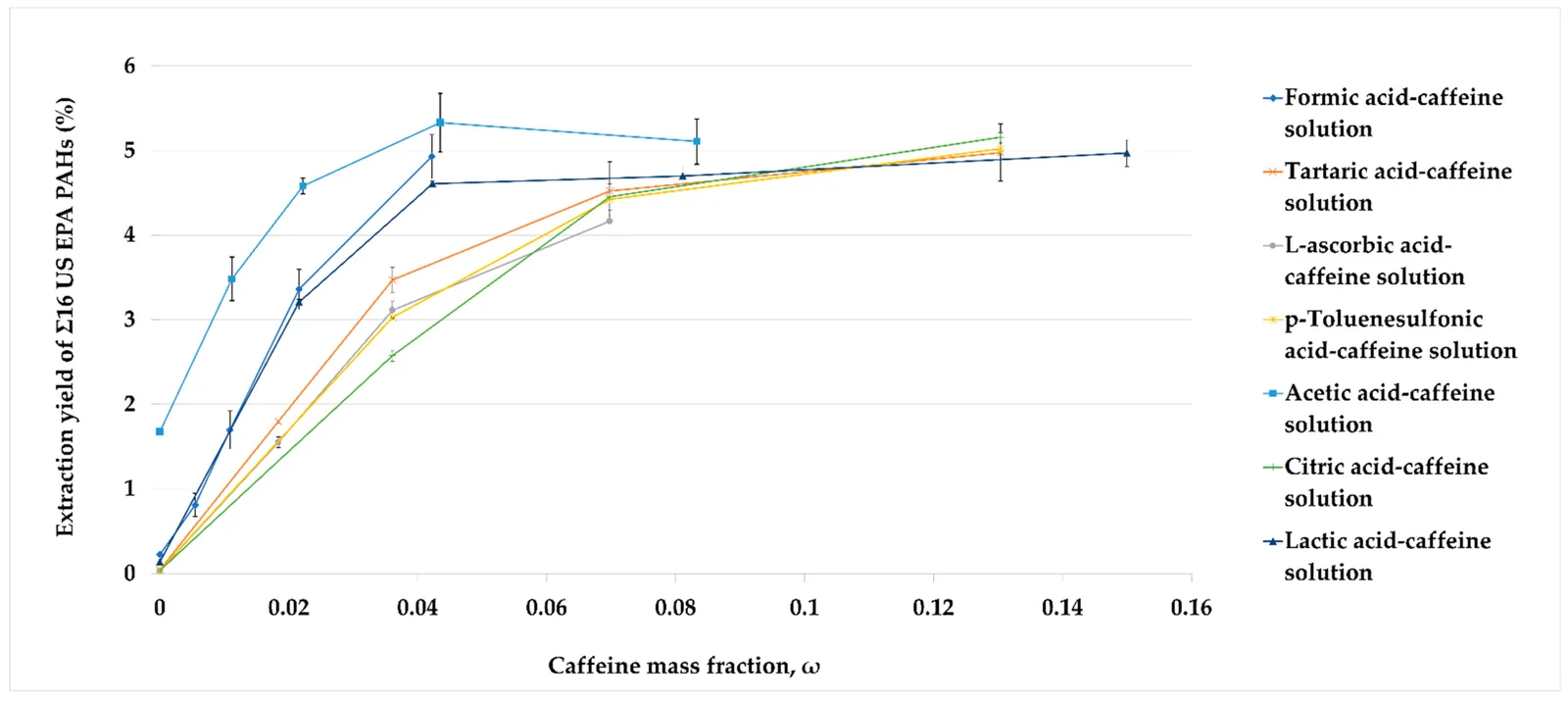
Effect of caffeine mass fraction on the EY (%) of PAHs using different aqueous acidic extraction systems. The graphs present the mean EY (%) values of PAHs as a function of caffeine mass fraction for the investigated aqueous acidic extraction systems. Error bars present the standard deviation of two independent measurements. The absence of error bars in some data indicates low standard deviation values.

The second set of experiments included the determination of the PAH EY values and a comparative analysis of the most promising extraction agents selected from the first set of experiments, with particular emphasis on the effect of varying the caffeine mass fraction in solutions containing the same organic acid. An example of a GC-FID chromatogram of a WRT extract is shown in Figure S1, while an example of the occurrence of the 16 US EPA priority PAHs in aqueous extracts obtained from WRT chips is presented in the Supplementary Materials (Table S1). The control systems described in Table 1 (Section 3) were used as reference systems for evaluating the effect of caffeine on PAH extraction. Based on the results of the initial screening, seven representative extraction media, S2, S5, S6, S8, S9, S10, and S12, were selected for further investigation. The selection was based on their extraction performance while ensuring a representative range of acid strengths, molecular structures, and physicochemical properties. Particular emphasis was placed on the citric, tartaric, ascorbic, and lactic acids because of their reported biodegradability and, for several of them, bio-based origin. Acetic acid, formic acid, and p-toluenesulfonic acid were retained as chemically distinct reference systems. This selection allowed a systematic evaluation of the influence of the caffeine mass fraction across chemically diverse extraction systems. The control solutions were supplemented with caffeine at different mass fractions, and the corresponding PAH EY values obtained strictly by static soaking for 24 h at room temperature are presented in Figure 2. The figure combines the EY profiles of all investigated aqueous acidic extraction systems in a single plot, while the individual systems are identified in the legend.

Most caffeine-free systems exhibited very low extraction yields, confirming the limited ability of acidic aqueous media alone to solubilize hydrophobic PAHs. Following caffeine addition, the extraction yields increased markedly, reaching values of approximately 4–5.3% for most systems. The observed differences in PAH EY values can be primarily attributed to the presence and mass fraction of caffeine rather than to the minor changes in the composition of the aqueous organic acid solution. The observed nonlinear dependence between the caffeine mass fraction and extraction yield is characterized by a pronounced increase at lower caffeine concentrations, followed by a gradual decrease in the slope of the extraction curves, indicating the progressive establishment of caffeine-mediated hydrotropic solubilization. The individual behavior of the investigated systems is discussed below.

The lactic acid-caffeine system exhibited a typical hydrotropic response. The caffeine-free control solution (S12) showed a low extraction yield of 0.1363%, whereas caffeine addition increased the yield to 3.2078% at a caffeine mass fraction of 0.0216 and further to 4.97% at the highest investigated caffeine mass fraction (0.150). Compared with the caffeine-free system, the extraction yield increased by approximately 23.5-, 33.8-, 34.5-, and 36.5-fold at caffeine mass fractions of 0.0216, 0.0423, 0.0811, and 0.150, respectively. Most of the improvement was achieved at caffeine concentrations up to approximately 0.04, after which only minor increases were observed, indicating an approach toward a plateau. A similar behavior was observed for the citric acid caffeine system. The caffeine-free citric acid solution (S10) exhibited a very low extraction yield (0.0368%), while caffeine addition increased the extraction yield up to approximately 5.16%. The enhancement factors reached approximately 70-, 121-, and 140-fold at caffeine mass fractions of 0.0361, 0.0697, and 0.1304, respectively. The pronounced increase at lower caffeine concentrations followed by a reduced rate of improvement indicates that the system approached its maximum solubilization performance within the investigated range. The tartaric acid caffeine system showed the same general trend. The caffeine-free solution (S6) extracted only 0.0435% of the Σ16 US EPA PAHs, whereas the addition of caffeine increased the yield to approximately 5%. The extraction yield increased approximately 41-, 80-, 104-, and 114-fold at caffeine mass fractions of 0.0184, 0.0361, 0.0698, and 0.1304, respectively. A plateau region was approached at caffeine mass fractions around 0.07, suggesting that further increases in the caffeine concentration provided limited additional improvement. The L-ascorbic acid caffeine system also exhibited a strong dependence on the caffeine concentration. The extraction yield increased from 0.0353% in the absence of caffeine (S8) to 1.5478%, 3.1126%, and 4.1652% at caffeine mass fractions of 0.0184, 0.0361, and 0.0698, corresponding to approximately 44-, 88-, and 118-fold increases, respectively. Unlike the lactic, citric, and tartaric acid systems, no clear plateau was reached within the investigated concentration range, although the reduction in the slope of the curve indicates progressive saturation of the hydrotropic effect. The p-TsOH caffeine system exhibited a comparable trend. The caffeine-free system (S9) showed a low extraction yield of 0.0405%, while caffeine addition increased the yield continuously, reaching 5.0233% at the highest investigated caffeine mass fraction (0.1304). The corresponding enhancement factors were approximately 75-, 109-, and 124-fold at caffeine mass fractions of 0.0361, 0.0697, and 0.1304, respectively. Although a clear plateau was not reached, the decreasing slope at higher caffeine concentrations indicates that the system was approaching its maximum extraction performance. The acetic acid caffeine system showed a different behavior compared with the previously discussed systems. The caffeine-free acetic acid solution (S2) exhibited a substantially higher extraction yield (1.6738%), indicating a greater intrinsic extraction capability of this medium. Consequently, the relative enhancement after caffeine addition was lower, with a maximum increase of approximately 3.19-fold. The extraction yield increased to 5.3313% at a caffeine mass fraction of 0.0435, representing the highest yield among all investigated systems. However, further caffeine addition did not improve extraction and resulted in a slight decrease. This indicates the presence of an optimum caffeine/acetic acid ratio under the applied experimental conditions, beyond which additional caffeine does not provide further enhancement. The formic acid caffeine system showed intermediate behavior between the highly efficient acetic acid system and the weakly extracting acidic systems. The caffeine-free formic acid solution (S5) exhibited an extraction yield of 0.2234%, which was considerably higher than most other caffeine-free organic acid systems but lower than acetic acid. Caffeine addition progressively increased the extraction yield from 0.2234% to 0.8106%, 1.6940%, 3.3581%, and 4.9278% at caffeine mass fractions of 0.0055, 0.0109, 0.0216, and 0.0422, respectively. These values correspond to approximately 3.6-, 7.6-, 15.0-, and 22.1-fold increases compared with the caffeine-free system. The continuous increase without reaching a plateau indicates that the maximum extraction capability of this system was not achieved within the investigated concentration range.

To evaluate the influence of the organic acid type independently of the caffeine concentration, the caffeine-containing systems were compared at identical caffeine mass fractions. At a caffeine mass fraction of 0.0361, the extraction yields ranged from 3.0283% to 3.4714%. The highest yield was obtained for the citric acid caffeine system (3.4714%), followed by the tartaric acid caffeine and L-ascorbic acid caffeine systems (both 3.1126%), while the p-TsOH caffeine system showed the lowest value (3.0283%). The extraction performance followed the order: citric acid-caffeine > tartaric acid-caffeine ≈ ascorbic acid-caffeine > p-TsOH-caffeine. At a higher caffeine mass fraction of 0.0698, the extraction yields became even more similar, ranging from 4.1652% to 4.5226%. The observed order was: citric acid-caffeine > p-TsOH-caffeine > tartaric acid-caffeine ≈ ascorbic acid-caffeine. The relatively small differences among systems at identical caffeine concentrations indicate that the caffeine concentration has a dominant influence on PAH extraction, while the effect of the organic acid type becomes less pronounced after caffeine addition. When systems with similar caffeine mass fractions close to 0.04 were compared, the highest extraction yield was obtained for the acetic acid caffeine system (5.3313%), followed by formic acid-caffeine (4.9278%) and lactic acid caffeine (4.6075%). The approximate order of extraction performance was: acetic acid-caffeine > formic acid-caffeine > lactic acid-caffeine > citric acid-caffeine > tartaric acid-caffeine ≈ ascorbic acidcaffeine > p-TsOH-caffeine. Comparison of the maximum extraction yields achieved within the investigated concentration ranges showed that the highest PAH extraction yield was obtained for the acetic acid-caffeine system (5.3313%), followed by citric acid-caffeine (5.1584%) and p-TsOH-caffeine (5.0233%). The tartaric acid-caffeine, lactic acid-caffeine, and formic acid-caffeine systems achieved similar maximum yields close to 5%, whereas the ascorbic acid-caffeine system exhibited the lowest maximum extraction yield. Based on the maximum extraction yields, the investigated systems can be ranked as follows: acetic acid-caffeine > citric acid-caffeine > p-TsOH-caffeine ≈ tartaric acid-caffeine ≈ lactic acid-caffeine ≈ formic acid-caffeine > ascorbic acid-caffeine. Overall, the combined analysis of extraction profiles demonstrates that caffeine addition substantially reduces the differences among the investigated aqueous acidic systems. While caffeine-free systems exhibited markedly different extraction capabilities, caffeine-containing systems converged toward a relatively narrow range of extraction yields (approximately 4–5.3%). This convergence confirms that caffeine-mediated hydrotropic solubilization is the dominant factor governing PAH extraction, whereas the organic acid mainly influences the baseline extraction capability and the magnitude of caffeine-assisted enhancement.

In our previous study [33], the extraction of PAHs from creosote-impregnated wooden railroad ties using conventional organic solvents demonstrated that dichloromethane exhibited the highest extraction capability, reaching PAH recovery values of 7.89% in Soxhlet extraction and 7.50% in solid-liquid batch extraction. The high performance of dichloromethane can be attributed to its favorable solvation properties toward hydrophobic aromatic compounds. However, despite its extraction capability, the use of chlorinated organic solvents presents environmental and operational limitations associated with solvent toxicity and volatility. In comparison, the aqueous organic acid caffeine systems investigated in the present study achieved PAH extraction yields of up to approximately 5.3% under mild extraction conditions. Although these values are slightly lower than those obtained with dichloromethane, the difference in extraction performance should be considered together with the substantial differences in solvent characteristics. The developed systems rely on water as the primary extraction medium and utilize caffeine as a hydrotropic agent to enhance the apparent solubility of hydrophobic PAHs.

### 2.3. The Third Set of Tests: Assessment of Sequential Batch Extraction for PAH Extraction from WRTs

**Figure 3:**
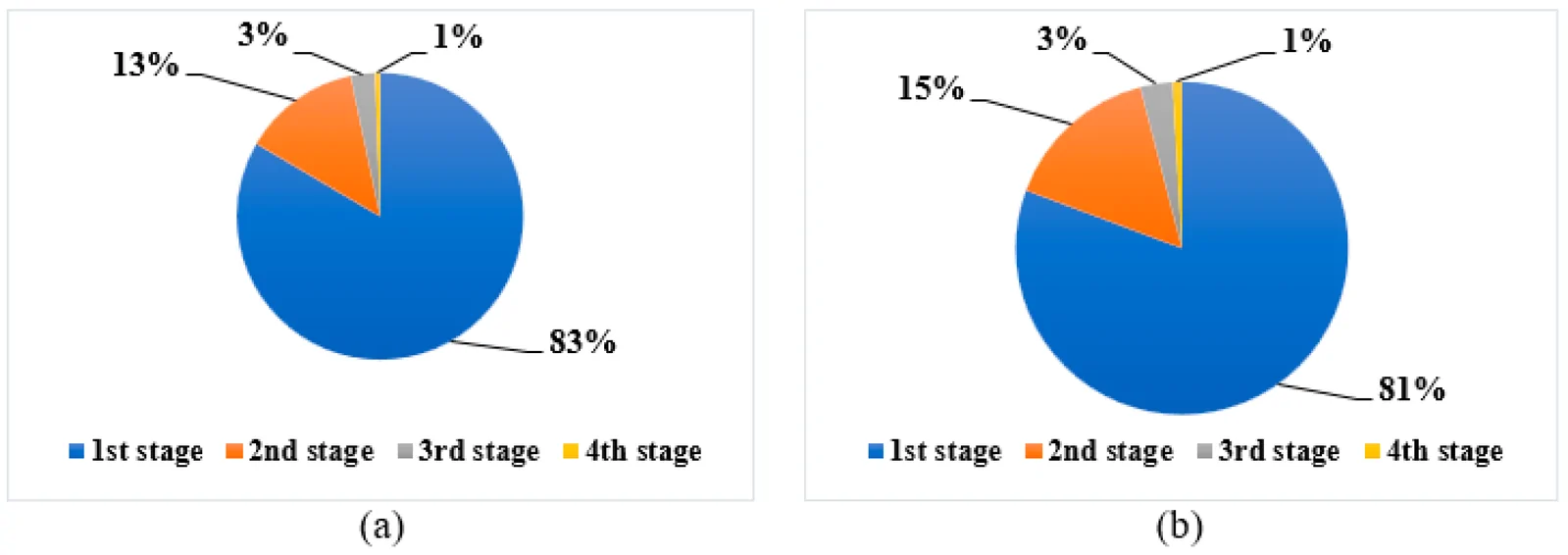
Contribution of individual extraction stages to the total PAH removal during multistage batch extraction for (**a**) S10C_0.1304_ and (**b**) S6C_0.1304_ aqueous agents.

The third set of experiments investigated multistage batch extraction using a fresh portion of the extraction agent at each extraction stage. The extraction procedure consisted of four consecutive stages, each lasting 24 h. At the end of each stage, the suspension was subjected to gravity filtration, after which a fresh portion of the extraction agent was added to the flask containing the previously extracted wood matrix. The overall extraction time was 96 h. The results of the conducted experiments are presented in Figure 3 and further discussed in terms of extraction performance.

Based on the results obtained in the second set of experiments, two aqueous extraction systems were selected for the multistage extraction study: 30 wt% citric acid in deionized water with caffeine mass fraction, ω = 0.1304 (S10C_0.1304_), and 30 wt% tartaric acid in deionized water with caffeine mass fraction, ω = 0.1304 (S6C_0.1304_). These systems were selected because they exhibited the highest extraction yields among the investigated citric acid caffeine and tartaric acid caffeine systems, respectively.

The multistage extraction approach was employed to investigate whether repeated extraction with fresh portions of the selected hydrotropic systems could further increase PAH recovery from the wood matrix or whether, as suggested by the preceding experiments, the extraction yield would converge toward a limiting value of approximately 5% under the investigated conditions.

The results of the multistage batch extraction for S10C_0.1304_ are presented in Figure 3a. The total amount of extracted PAHs was 47,064.78 µg/g. The first extraction stage showed the highest contribution, with 39,276.50 µg/g of extracted PAHs, corresponding to 83.45% of the total recovered amount. The second extraction stage resulted in an additional recovery of 6238.44 µg/g (13.26%), whereas the third and fourth stages contributed 1285.35 µg/g (2.73%) and 264.50 µg/g (0.56%), respectively. The obtained results demonstrate a progressive decrease in extraction performance with each subsequent extraction stage, indicating that most of the extractable PAHs were removed during the first stage of the process. The contribution of the fourth extraction stage was negligible, accounting for only 0.56% of the total PAH recovery.

The multistage extraction results for S6C_0.1304_ are presented in Figure 3b. The total amount of extracted PAHs was 46,416.52 µg/g. The first extraction stage yielded 37,444.11 µg/g of PAHs, corresponding to 80.7% of the total recovered amount. The second extraction stage resulted in an additional recovery of 7100.11 µg/g (15.3%), while the third and fourth stages contributed 1454.40 µg/g (3.1%) and 417.91 µg/g (0.9%), respectively. These results demonstrate that the amount of PAHs extracted decreased markedly with each successive extraction stage, indicating progressive depletion of the readily extractable PAH fraction from the wood matrix. Although each additional extraction stage contributed a smaller amount of PAHs, the use of fresh extraction agent enabled further removal of residual compounds that remained inaccessible after the previous stage. The decreasing contribution of successive stages is consistent with the gradual depletion of easily desorbable PAHs and the increasing resistance to mass transfer of the remaining compounds retained within the wood structure.

To further investigate the extraction behavior, the total PAH recovery obtained by single-stage and multistage batch extraction was compared for the S10C_0.1304_ and S6C_0.1304_ extraction systems. For S10C_0.1304_, the single-stage extraction in the second set of tests resulted in a PAH recovery of 51,584.48 ± 557.28 µg/g (n = 2), while the cumulative amount obtained after four consecutive extraction stages was 47,064.7782 ± 631.3409 µg/g (n = 2). Similarly, for S6C_0.1304_, the single-stage extraction yielded 49,760.97 ± 3390.45 µg/g (n = 2), compared with 46,416.5230 ± 201.7876 µg/g (n = 2), obtained by multistage extraction.

The fact that the cumulative recovery after four extraction stages did not exceed the recovery obtained in the single-stage experiment suggests that, under the investigated conditions, the extraction process approaches a limiting value. However, the present results do not allow the origin of this apparent limitation to be unequivocally established. It may be related to the actual amount of PAHs that is extractable from the investigated WRT sample, limited accessibility of creosote-containing regions within the heterogeneous wood matrix, mass transfer limitations, or other interactions between the PAHs, wood matrix, and extraction system. Therefore, the observed convergence toward approximately 5% yield should be regarded as an indication of an apparent extraction limit rather than as definitive evidence that 5% represents the maximum PAH content of the sample.

The differences between the single-stage and multistage recoveries should also be interpreted with caution because the experiments were conducted using separate sample portions. Despite homogenization, the heterogeneous distribution of creosote within the WRT matrix may result in differences in the PAH content and accessibility among individual sample portions. Consequently, the present experiment is considered exploratory and provides evidence for diminishing extraction returns and an apparent recovery plateau, while further experiments would be required to distinguish between limitations arising from sample composition, matrix accessibility, and the extraction process itself.

### 2.4. The Fourth Set of Tests: Effect of Extraction Time on PAH Extraction Using Caffeine-Assisted Aqueous Extraction Systems

**Figure 4:**
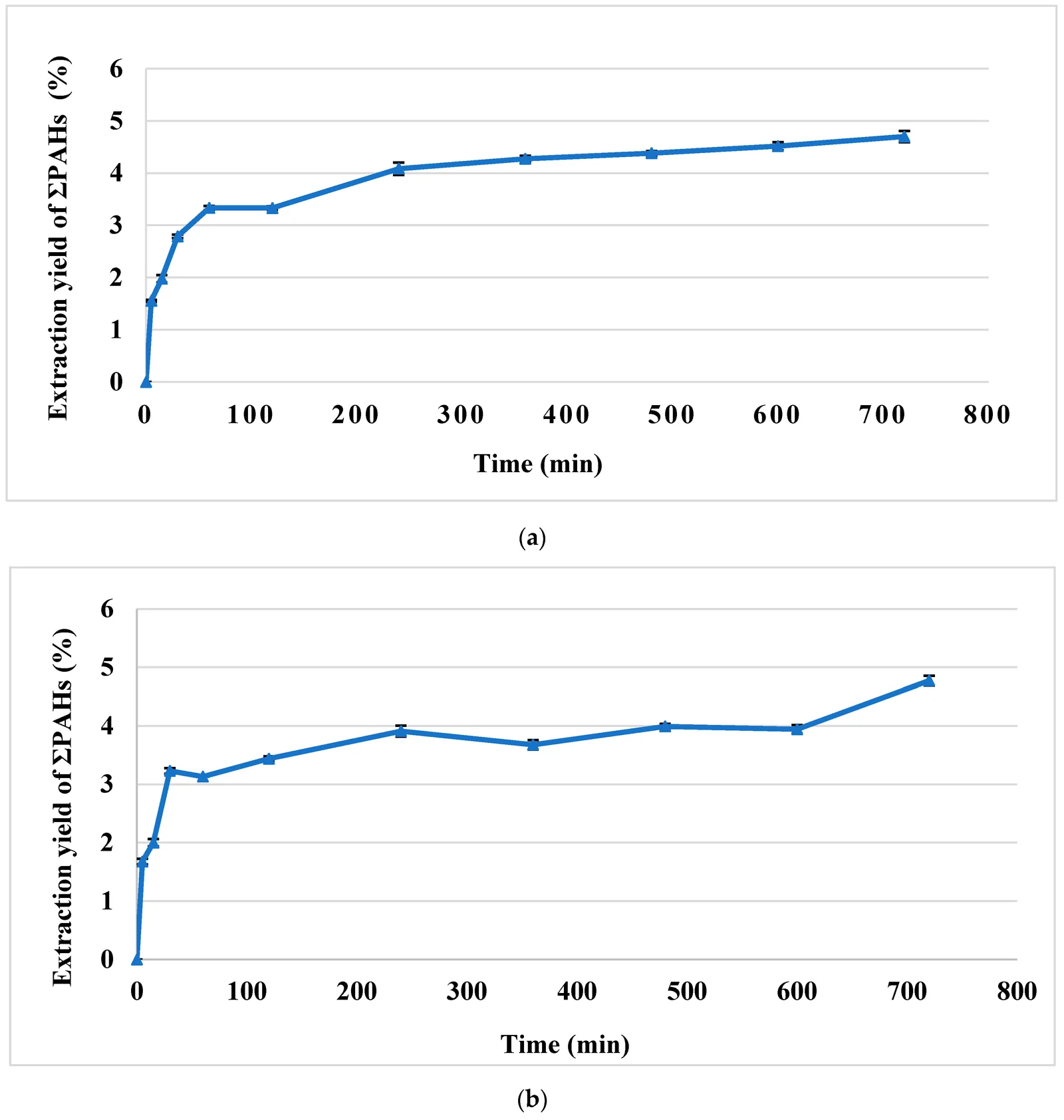
Extraction kinetics of PAHs from creosote-impregnated wood using caffeine-assisted aqueous extraction systems (**a**) S10C_0.1304_ and (**b**) S6C_0.1304_. Error bars represent the standard deviation (SD) of two independent measurements (n = 2). The absence of error bars in some data indicates low standard deviation values.

The fourth set of experiments involved batch extraction performed over a period ranging from 0 min to 720 min, using 11 selected extraction time intervals. Two aqueous extraction agents identified as the most promising in the previous set of experiments were selected for this study: 30 wt% citric acid in deionized water with caffeine mass fraction, ω= 0.1304 (S10C_0.1304_), and 30 wt% tartaric acid in deionized water with caffeine mass fraction, ω = 0.1304 (S6C_0.1304_). The effect of the extraction time on PAH yield using the S10C_0.1304_ and S6C_0.1304_ extraction systems is presented in Figure 4.

The extraction process using the S10C_0.1304_ extraction system showed a rapid increase in PAH yield during the initial extraction period, followed by a gradual increase with prolonged extraction time. After 5 min of extraction, the PAH yield reached 1.5553%, increasing to 1.9776% and 2.7888% after 15 and 30 min, respectively. Further extension of the extraction time resulted in an increase in extraction yield, reaching 3.3322% after 60 min. Between 60 and 120 min, the extraction yield remained nearly constant (3.3322 and 3.3308%, respectively), indicating a temporary equilibrium or slower mass transfer process. Prolonged extraction resulted in a further gradual increase in PAH recovery, reaching 4.7004% after 720 min. The extraction rate was highest during the initial extraction period, reaching 0.3111%/min within the first 5 min. The most pronounced transition in the extraction behavior occurred between 5 and 60 min, when the extraction rate decreased significantly, indicating the transition from rapid desorption of easily accessible PAHs to a slower extraction stage. After 60 min, the extraction rate further decreased, reaching approximately 10^−3^%/min during prolonged extraction, which suggests that the process became increasingly controlled by diffusion and mass transfer limitations within the wood matrix. The observed extraction behavior indicates an initial rapid release of readily extractable PAHs, followed by a slower extraction stage associated with the gradual depletion of accessible PAHs and the release of more strongly retained compounds.

The extraction process using the S6C_0.1304_ extraction system showed a rapid increase in PAH yield during the initial extraction period, followed by a slower and less uniform increase with prolonged extraction time. After 5 min of extraction, the PAH yield reached 1.6773%, increasing to 2.0003% and 3.2297% after 15 and 30 min, respectively. After 60 min, a slight decrease in extraction yield was observed (3.1317%), which may be attributed to the heterogeneous distribution of PAHs within the wood matrix and the inherent variability associated with the extraction of a complex solid sample. Further extraction resulted in a gradual increase in PAH recovery, reaching 3.4392% after 120 min, 3.9106% after 240 min, and 4.7726% after 720 min. Although minor fluctuations in extraction yield were observed during the prolonged extraction period, the overall trend indicated continuous release of PAHs from the wood matrix. The extraction rate was highest during the initial extraction period, reaching 0.3355%/min within the first 5 min. After this initial stage, the extraction rate decreased considerably; however, a temporary increase was observed between 15 and 30 min, where the extraction rate reached 0.0820%/min, indicating continued desorption of the readily accessible PAH fraction. The most pronounced transition in the extraction behavior occurred within the first 60 min, when the process shifted from a rapid extraction stage to a slower stage characterized by reduced extraction rates and minor variations in recovery. At prolonged extraction times, the extraction rate remained considerably lower (generally below 0.01%/min), suggesting that the extraction process became increasingly controlled by diffusion and mass transfer limitations within the wood structure. The observed extraction profile indicates an initial rapid removal of easily accessible PAHs, followed by a slower extraction stage associated with the gradual depletion of the readily extractable fraction and the release of more strongly retained PAHs from the creosote-impregnated wood matrix.

## 3. Materials and Methods

### 3.1. Materials

A waste creosote-treated railroad tie, obtained from a landfill disposal site in Serbia after the end of its service life, was used throughout the study as the test material. The tie was mechanically processed prior to the extraction experiments. Deionized water was used for the preparation of all aqueous extraction systems. The investigated organic acids included acetic acid (≥99.7%, Merck, Darmstadt, Germany), citric acid monohydrate (≥99.5%, Merck, Darmstadt, Germany), formic acid (85%, Merck, Darmstadt, Germany), lactic acid (85%, Sigma-Aldrich, St. Louis, MO, USA), oxalic acid dihydrate (≥99.5%, Merck, Darmstadt, Germany), succinic acid (≥99%, Sigma-Aldrich, St. Louis, MO, USA), tartaric acid (≥99.5%, Merck, Darmstadt, Germany), L-ascorbic acid (≥99%, Sigma-Aldrich, St. Louis, MO, USA), and p-toluenesulfonic acid monohydrate (≥98.5%, Carl Roth GmbH + Co. KG, Karlsruhe, Germany). Caffeine (≥99%, Carlo Erba Reagents, Val de Reuil, France) was used as a hydrotropic agent. n-Hexane (HPLC grade, Carlo Erba Reagents, Val de Reuil, France), sodium chloride (≥99.5%, Lach-Ner, s.r.o., Neratovice, Czech Republic), and anhydrous sodium sulfate (Lach-Ner, s.r.o., Neratovice, Czech Republic) were used during the liquid–liquid extraction procedure. Unless otherwise stated, all chemicals were of analytical grade and used as received without further purification. A certified PAH-Mix 14 standard solution (Dr. Ehrenstorfer, LGC, Augsburg, Germany), containing 2000 μg mL^−1^ of each component in an acetone/benzene mixture, was used to prepare calibration standards for the quantitative determination of the 16 US EPA priority PAHs.

### 3.2. Instrumental Analysis, Identification and Quantification of 16 US EPA PAHs

The quantitative determination of the Σ16 US EPA PAHs was performed using gas chromatography coupled with flame ionization detection (GC-FID). Analyses were carried out using a Shimadzu Nexis GC-2030 gas chromatograph with LabSolutions software, Version 5.97, Shimadzu Corporation (Nakagyo-ku, Kyoto, Japan), equipped with an Autosampler AOC-20i, split/splitless injection unit (SPL), capillary column Thermo TG-4MS, length 30 m × 0.25 mm I.D, 0.25 µm thick film (Thermo Fisher Scientific, Waltham, MA, USA) and a flame ionization detector. The GC temperature program of the method for the detection of PAHs is as follows: the initial temperature of the column is 40 °C and the hold time is 4 min. Then, the temperature increases at a rate of 10 °C/min until it reaches 300 °C. The hold time at this temperature is 15 min. The entire run lasts 45 min. Detection of analytes was performed with a flame ionization detector (FID) at a temperature of 315 °C. The identification and quantification of PAHs were performed using calibration standards prepared from a certified standard mixture. External calibration was used and calibration plots were achieved using ten different concentration levels of the 16 PAHs. PAH-Mix 14 standard solution (Dr. Ehrenstorfer, LGC, Augsburg, Germany), containing 18 PAHs, including the 16 US EPA priority PAHs and 1-methylnaphthalene and 2-methylnaphthalene, was used as the reference standard. Only the 16 US EPA priority PAHs were considered in the quantitative analysis. Satisfactory coefficients of determination (0.9973–0.9999) were obtained for all the 16 PAHs in the range of 0.5–50.0 μg/mL and three different calibration curves. The 16 individual US EPA priority PAHs were identified and quantified separately by GC-FID, and the concentration of each compound was determined using its corresponding calibration curve. The minimum concentration of analyte that can be quantified by the method is referred to as the limit of quantification (LOQ). The extraction yield of the Σ16 US EPA PAHs was calculated based on the mass of PAHs extracted relative to the initial mass of the analyzed waste wood sample and expressed as a percentage. Statistical calculations and graphical representations were performed using the mean values obtained from duplicate experiments (n = 2). The total PAH content was determined independently for two test samples and the standard deviation was calculated as the standard deviation based on a sample. All results are expressed as percentages with associated standard deviations (mean ± SD). The standard deviation of the summed values was calculated assuming independent measurements. The standard deviation of the mean was obtained using the combined standard deviation, considering individual measurement uncertainties.

### 3.3. Preparation of Aqueous Extraction Systems

Aqueous extraction systems were prepared using deionized water and selected water-soluble organic acids, namely acetic, citric, formic, lactic, oxalic, p-toluenesulfonic, tartaric, and ascorbic acids. The acids were dissolved in deionized water at different concentrations depending on the experimental design. Caffeine was subsequently added to selected acid solutions as a hydrotropic agent. For several extraction systems, the amount of dissolved caffeine was limited by its solubility under the experimental conditions. The composition of investigated aqueous extraction systems, together with their measured pH values, is summarized in Table 1 and in the Supplementary Materials, Table S2. Caffeine at the highest mass fractions was dissolved in the organic acid solutions until saturation was reached, with brief heating to 50 °C to facilitate dissolution or the highest caffeine mass fraction was selected based on the maximum amount of caffeine that could be dissolved in the organic acid solution without exceeding the saturation limit.

### 3.4. Sample Preparation

A waste creosote-treated WRT, obtained from a landfill disposal site in Serbia after the end of its service life, was used throughout the study. The entire railway tie was mechanically shredded using a stainless-steel milling machine to obtain wood chips with average dimensions of approximately 16 × 0.5 × 0.3 mm (length × width × thickness). The reduced particle size increases the contact area between the wood matrix and the extraction medium, thereby facilitating mass transfer during extraction. Following shredding, the entire sample was thoroughly homogenized to ensure uniform distribution of both the wood matrix and creosote-derived contaminants. Experimental portions were subsequently collected from the homogenized material for all extraction experiments.

Following extraction, the aqueous extract was separated from the wood matrix by gravity filtration through fast flow cellulose filter paper. The filtrate was transferred to a separatory funnel, and 200 mL of a 2 wt% aqueous sodium chloride solution was added to reduce the solubility of PAHs in the aqueous phase. The aqueous phase was subsequently extracted twice with 80 mL portions of n-hexane using liquid–liquid extraction. The combined n-hexane extracts were dried over anhydrous sodium sulfate prior to instrumental analysis. Liquid samples in n-hexane were prepared by filtering aliquots of extract through membrane nylon filters (Whatman Uniflo^®^, Cytiva, Marlborough, MA, USA), without the cleaning-up procedure. Visual appearance of WRT chips before and after extraction under different extraction conditions is presented in the Supplementary Materials in Figure S2 and WRT chips during the sample preparation in Figure S3.

The dry matter content of creosote-treated WRT chips was determined gravimetrically. Prior to analysis, the WRT was mechanically reduced into chips and homogenized to obtain representative test samples. Approximately 2 g of each of two test samples was weighed and the initial mass was recorded. The samples were dried in a laboratory oven (Binder Model FD-S 115, Tuttlingen, Germany) at 105 ± 2 °C until constant mass was achieved, with intermediate weighing performed after cooling in a desiccator. The drying–cooling–weighing cycle was repeated until the difference between two consecutive measurements did not exceed 0.1%. The dry matter content was then calculated as the ratio of the constant dry mass to the initial mass and expressed as a percentage.

### 3.5. Experimental Design

Four sets of experiments were designed to evaluate the applicability of aqueous extraction systems for the extraction of polycyclic aromatic hydrocarbons from creosote-treated WRTs. The first set involved single-stage solid–liquid batch extraction without mechanical agitation using aqueous solutions containing different organic acids, with and without caffeine. The second set focused on the influence of the caffeine concentration on the extraction performance of selected aqueous systems. The third set consisted of multistage solid–liquid batch extraction using the most promising aqueous systems identified in the previous experiments. The fourth set involved the monitoring of the extraction kinetics of selected aqueous extraction agents over time. All WRT test portions were prepared from the homogenized material obtained after shredding of the waste railway tie. The extraction performance of the investigated aqueous systems was evaluated based on the extraction yield (EY) of the sum of the 16 US EPA priority PAHs. The effect of caffeine addition on PAH extraction was assessed by comparing the extraction yields obtained with caffeine-free systems and their corresponding caffeine-containing counterparts.

#### 3.5.1. The First Set of Tests: Effect of Caffeine Addition on PAH Extraction Performances from Creosote-Treated Wood in Different Extraction Media

The first set of experiments was designed to investigate the influence of caffeine addition on the extraction performance of aqueous systems containing different organic acids. Aqueous extraction media were prepared using deionized water and selected water-soluble organic acids: acetic acid, citric acid, formic acid, lactic acid, oxalic acid, succinic acid, tartaric acid, ascorbic acid, and p-toluenesulphonic acid. For each extraction medium, corresponding systems with and without caffeine were prepared to evaluate the effect of caffeine addition on PAH extraction performance. Different concentrations of organic acids and different amounts of caffeine were investigated. The detailed composition of S0–S13 and the corresponding caffeine-containing aqueous systems’ is presented in Table 2.

The corresponding caffeine-containing systems (S0C–S13C) were prepared by adding caffeine to the respective extraction media. In systems S1C and S13C, the caffeine concentration was limited by its solubility under the applied experimental conditions. Caffeine mass fraction represents the mass fraction of caffeine in the total extraction solution. Extraction experiments were performed using homogenized creosote-treated WRT samples. A mass of 1.00 g of the WRT sample was contacted with 40.0 mL of the prepared aqueous extraction medium in a 300 mL Erlenmeyer flask. The samples were immersed in the extraction medium and maintained at room temperature (25 °C) for 24 h without mechanical agitation. After extraction, the aqueous phase was separated from the solid wood matrix by gravity filtration and subjected to sample preparation prior to GC-FID analysis, as described in Section 3.4.

#### 3.5.2. The Second Set of Tests: Extraction with Organic Acid-Caffeine Solutions of Different Caffeine Mass Fractions

The second set of experiments was designed to investigate the influence of the caffeine concentration on the extraction performance of selected aqueous organic acid systems. Based on the results of the first set of experiments, selected organic acid solutions were used as extraction media, and different amounts of caffeine were added to obtain extraction systems with different caffeine mass fractions. The investigated extraction systems were prepared by dissolving predefined amounts of caffeine in aqueous solutions of selected organic acids. The caffeine mass fraction (ω) was varied by changing the amount of added caffeine while maintaining the composition of the corresponding organic acid solution. The detailed composition of the investigated extraction systems and caffeine mass fractions is presented in the Supplementary Materials, Table S2. Extraction experiments were performed using 1.00 g of homogenized creosote-treated WRT sample and 40.0 mL of the prepared organic acid-caffeine extraction system in a 300 mL Erlenmeyer flask. The samples were kept immersed in the extraction medium at room temperature (25 °C) for 24 h without mechanical agitation. After extraction, the solid and liquid phases were separated by gravity filtration, and the obtained extracts were subjected to sample preparation prior to GC-FID analysis, as described in Section 3.4. The extraction performance of the investigated systems was evaluated based on the extraction yield of the 16 US EPA PAHs as a function of the caffeine mass fraction.

#### 3.5.3. The Third Set of Tests: Assessment of Sequential Batch Extraction for PAH Extraction from WRTs

The third set of experiments was designed to evaluate the performance of sequential batch extraction for the extraction of PAHs from creosote-treated WRTs. The extraction systems selected for this investigation were chosen based on their performance in the previous experiments. Two aqueous organic acid-caffeine extraction systems were investigated in a multistage extraction procedure. Sequential batch extraction was performed using fresh portions of the extraction medium in each extraction stage. In each stage, 1.00 g of homogenized WRT sample was contacted with 40.0 mL of the extraction system in a 300 mL Erlenmeyer flask. The samples were kept immersed in the extraction medium at room temperature (25 °C) for 24 h without mechanical agitation. After each extraction stage, the solid and liquid phases were separated by gravity filtration, and the aqueous extract was collected for further sample preparation prior to GC-FID analysis, as described in Section 3.4. A new portion of fresh extraction medium was added to the remaining solid residue for the subsequent extraction stage. The sequential extraction procedure was conducted in four consecutive stages. Successive extractions were achieved till the sum of the 16 US EPA PAHs was below the limit of quantification. The cumulative extraction performance was evaluated based on the extraction yield of the 16 US EPA PAHs obtained after each extraction stage and after the complete extraction sequence.

#### 3.5.4. The Fourth Set of Tests: Effect of Extraction Time on PAH Extraction Using Caffeine-Assisted Aqueous Extraction Systems

The fourth set of experiments was designed to investigate the influence of the extraction time on the extraction performance of the caffeine-assisted aqueous extraction systems. The selected extraction agents were chosen based on their performance in the previous experimental sets. The time dependence of PAH extraction was evaluated in order to monitor the extraction kinetics of the 16 US EPA PAHs from the creosote-treated WRTs. Extraction experiments were performed using 1.00 g of homogenized WRT sample and 40.0 mL of the selected caffeine-assisted aqueous extraction system in a 300 mL Erlenmeyer flask. The samples were kept immersed in the extraction medium at room temperature (25 °C) without mechanical agitation. Extraction was carried out for different time intervals, ranging from 0 min to 720 min. At each selected time point, the solid and liquid phases were separated by gravity filtration, and the obtained extracts were subjected to sample preparation prior to GC-FID analysis, as described in Section 3.4. The effect of the extraction time was evaluated based on the extraction yield of the 16 US EPA PAHs obtained at each time interval. The obtained data were used to assess the extraction kinetics and the time required to achieve effective extraction using the investigated caffeine-assisted aqueous systems.

## 4. Conclusions

The aim of this study was to develop and evaluate a novel caffeine-assisted aqueous extraction approach for the leaching of polycyclic aromatic hydrocarbons (PAHs) from creosote-treated waste wood. The results demonstrated that caffeine significantly enhanced PAH extraction in all investigated systems, although the magnitude of improvement depended on the initial extraction performance of the corresponding caffeine-free medium. The greatest relative enhancements were observed for extraction media exhibiting low initial extraction efficiencies, whereas systems with higher initial performance showed smaller relative improvements. The water–caffeine system achieved an extraction yield of 1.6886%, corresponding to approximately a 625-fold increase compared with water alone. Among the investigated systems, acetic acid-caffeine achieved the highest PAH extraction yield (5.3313%), followed by citric acid-caffeine (5.1584%) and p-TsOH-caffeine (5.0233%). Other systems showed maximum yields close to 5%, except ascorbic acid-caffeine, which showed the lowest yield. The present investigation reflects the PAH extraction from the specific WRT used in this study, but as the creosote treatment procedures of novel railway ties differ for various producers, resulting in different creosote uptakes, possible differences in the extraction yields could be expected.

A key finding of this work is that, despite the large differences in extraction yields among the caffeine-free systems, the addition of caffeine produced remarkably similar extraction yields for most investigated media. This behavior indicates that caffeine-induced hydrotropic solubilization became the predominant factor governing PAH extraction under the investigated conditions. Furthermore, increasing the caffeine concentration resulted in progressively higher extraction yields, confirming the concentration-dependent nature of its hydrotropic effect. The combination of caffeine with selected organic acids provided the highest extraction performance, indicating that the acidic medium promotes caffeine solubility and consequently enhances its hydrotropic action. For the two investigated extraction systems (30 wt% citric acid aqueous solution with caffeine and 30 wt% tartaric acid aqueous solution with caffeine), extraction was fastest during the initial 5 min, followed by a marked decrease in the extraction rate within the first 60 min, indicating a transition from rapid initial PAH desorption to slower extraction. The novelty lies in showing that water, despite its limited ability to solubilize hydrophobic PAHs, can be modified with caffeine and selected organic acids to enhance PAH extraction.

## Figures and Tables

**Table 1:** Extraction yield of Σ16 US EPA PAHs obtained using aqueous extraction agents without and with caffeine.

Aqueous Agent	EY of PAHs Without Caffeine (%)	EY of PAHs with Caffeine (%)
S0/S0C_0.0196_	0.0027 ± 0.0003	1.6886 ± 0.0401
S1	5.4907 ± 0.0317	-
S2/S2C_0.0435_	1.6738 ± 0.0264	5.3313 ± 0.3469
S3/S3C_0.0530_	0.2708 ± 0.0119	4.6127 ± 0.1449
S4/S4C_0.15_	3.4648 ± 0.1862	4.6796 ± 0.2613
S5/S5C_0.0423_	0.2234 ± 0.0217	4.9278 ± 0.2581
S6/S6C_0.0698_	0.0435 ± 0.0051	4.5226 ± 0.3486
S7/S7C_0.0698_	0.0256 ± 0.0008	4.6420 ± 0.0213
S8/S8C_0.0698_	0.0353 ± 0.0065	4.1652 ± 0.0110
S9/S9C_0.0698_	0.0405 ± 0.0019	4.4245 ± 0.1821
S10/S10C_0.0698_	0.0368 ± 0.0053	4.4531 ± 0.1534
S11/S11C_0.0698_	0.0281 ± 0.0027	4.2857 ± 0.3074
S12/S12C_0.0423_	0.1363 ± 0.0176	4.6075 ± 0.0341
S13	0.0121 ± 0.0007	-

Note: S0, deionized water; S1, 99.9% acetic acid; S2, 51.5 wt% acetic acid solution; S3, 33 wt% acetic acid solution; S4, 85% formic acid; S5, 42.5 wt% formic acid solution; S6, 30 wt% tartaric acid solution; S7, 15 wt% tartaric acid solution; S8, 15 wt% L-ascorbic acid solution; S9, 15 wt% p-toluenesulphonic acid monohydrate solution; S10, 30% citric acid in deionized water; S11, 15% citric acid in deionized water; S12, 42.5 wt% lactic acid solution; S13, 7.5% oxalic acid in deionized water. C denotes the corresponding caffeine-containing system with caffeine mass fraction in subscript.

**Table 2:** Composition of the control and caffeine-containing extraction systems.

Control System	Extraction Medium	Caffeine-Containing System	Caffeine Mass Fraction (ω)
S0	Deionized water	S0C_0.0196_	0.0196
S1	Concentrated acetic acid (99.9%)	S1C	Limited by solubility
S2	51.5 wt% acetic acid solution	S2C_0.0435_	0.0435
S3	33 wt% acetic acid solution	S3C_0.0530_	0.0530
S4	Concentrated formic acid (85%)	S4C_0.15_	0.1500
S5	42.5 wt% formic acid solution	S5C_0.0423_	0.0423
S6	30 wt% tartaric acid solution	S6C_0.0698_	0.0698
S7	15 wt% tartaric acid solution	S7C_0.0698_	0.0698
S8	15 wt% L-ascorbic acid solution	S8C_0.0698_	0.0698
S9	15 wt% p-toluenesulphonic acid monohydrate solution	S9C_0.0698_	0.0698
S10	30 wt% citric acid solution	S10C_0.0698_	0.0698
S11	15 wt% citric acid solution	S11C_0.0698_	0.0698
S12	42.5 wt% lactic acid solution	S12C_0.0423_	0.0423
S13	7.5 wt% oxalic acid solution	S13C	Limited by solubility

Note: S0–S13 denote the reference extraction systems, while SxC denotes the corresponding caffeine-containing system. The caffeine mass fraction (ω) represents the mass fraction of caffeine in the final extraction solution. For systems S1C and S13C, the maximum caffeine concentration was limited by caffeine solubility in the respective extraction medium.

## Data Availability

The original contributions presented in the study are included in the article; further inquiries can be directed to the corresponding authors.

## Supplementary Materials

The following supporting information can be downloaded at: https://www.mdpi.com/article/10.3390/molecules31173107/s1, Figure S1. GC-FID chromatogram of WRT extract in S9C0.0698; Table S1. Quantitative analysis of the 16 US EPA priority PAHs in the WRT test sample (S9C0.0698 extract) by GC-FID; Table S2: The caffeine mass fraction and EY of investigated aqueous extraction systems; Figure S2. Visual appearance of WRT chips before and after extraction under different extraction conditions: (**a**) WRT chips before extraction; (**b**) WRT chips after 24 h of extraction in S10C0.1304; and (**c**) WRT chips after 24 h of extraction in S4C0.15 and Figure S3. (**a**) WRT chips during the sample preparation and extraction process and (**b**) final extracts in n-hexane.
