# Supplementary material for: Novel Caffeine-Assisted Aqueous Extraction Strategy for Enhanced Solubilization of Polycyclic Aromatic Hydrocarbons from Creosote-Treated Waste Wood
Source: Molecules. 2026 Sep 4;31(17):3107. doi: 10.3390/molecules31173107 (PMC13566980; doi:10.3390/molecules31173107)
Supplement: Supplementary file 1 [file molecules-31-03107-s001.zip › molecules-4506223-supplementary.pdf]

Supplementary Materials

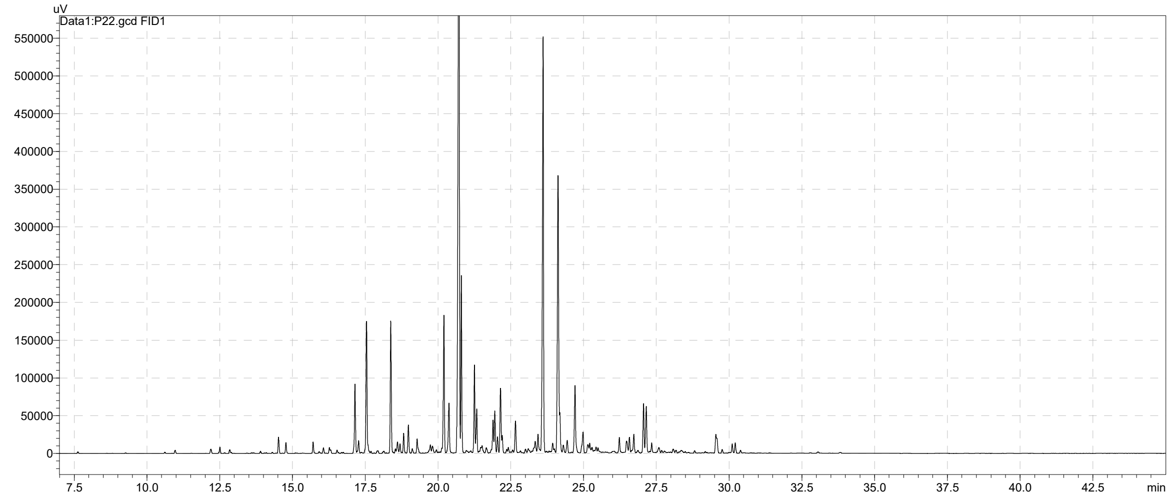

Figure S1. GC-FID chromatogram of WRT extract in $S9C_{0.0698}$.

Table S1. Quantitative analysis of the 16 US EPA priority PAHs in the WRT test sample ($S9C_{0.0698}$ extract) by GC-FID.

Serial number	Compound	Retention time, R_t (min)	C (µg/ml)
1	Naphthalene (Nap)	12.858	0.274
2	Acenaphthylene	-	<LOQ
3	Acenaphthene (Ace)	17.147	7.362
4	Fluorene (Fle)	18.378	14.137
5	Phenanthrene (Ph)	20.720	99.560
6	Anthracene (An)	20.802	17.338
7	Fluoranthene (Fla)	23.612	52.665
8	Pyrene (Py)	24.124	41.358
9	Benz(a)anthracene (B[a]A)	27.061	6.044
10	Crysene (Chr)	27.154	6.025
11	Benzo(b)fluoranthene (B[b]F)	29.547	3.692
12	Benzo(k)fluoranthene (B[k]F)	-	<LOQ
13	Benzo(a)pyrene (B[a]P)	30.214	1.249
14	Dibenzo(a,h)anthracene	-	<LOQ
15	Benzo(g,h,i)perylene	-	<LOQ
16	Indeno(1,2,3-c,d)pyrene (IP)	33.153	0.047

Note: The table presents an example of the occurrence of the 16 US EPA priority PAHs in aqueous extracts obtained from WRT ships; LOQ-limit of quantification.

Table S2. The caffeine mass fraction and EY of investigated aqueous extraction systems.

Aqueous agent	EY±SD (%) (n=2)	Caffeine mass fraction	pH
S0	0.0027±0.0003	0	5.63
$S0C_{0.0196}$	1.6886±0.0401	0.0196	5.92
S1	5.4907±0.0317	0	N/A
S1C	Limited by solubility	-	-

S2	1.6738±0.0264	0	1.93
S2C0.0112	3.4815±0.2577	0.0112	1.93
S2C0.0222	4.5814±0.0960	0.0222	1.98
S2C0.0435	5.3313±0.3469	0.0435	2.11
S2C0.0833	5.1085±0.2677	0.0833	2.15
S3	0.2708±0.0119	0	2.05
S3C0.0530	4.6127±0.1449	0.0530	2.12
S4	3.4648±0.1862	0	N/A
S4C0.15	4.6796±0.2613	0.15	N/A
S5	0.2234±0.0217	0	N/A
S5C0.0055	0.8106±0.1367	0.0055	N/A
S5C0.0109	1.6940±0.2254	0.0109	N/A
S5C0.0216	3.3581±0,2375	0.0216	N/A
S5C0.0423	4.9278±0,2581	0.0422	N/A
S6	0.0435±0,0051	0	1.38
S6C0.0184	1.7955±0.0170	0.0184	1.40
S6C0.0361	3.4714±0.1448	0.0361	1.44
S6C0.0698	4.5226±0.3486	0.0698	1.60
S6C0.1304	4.9761±0.3390	0.1304	1.64
S7	0.0256±0.0008	0	1.41
S7C0.0698	4.6420±0.0213	0.0698	1.73
S8	0.0353±0.0065	0	1.59
S8C0.0184	1.5478±0.0625	0.0184	1.62
S8C0.0361	3.1126±0.1008	0.0361	1.74
S8C0.0698	4.652±0.0110	0.0698	1.90
S9	0.0405±0.0019	0	0.88
S9C0.0361	3.0283±0.0033	0.0361	0.89
S9C0.0698	4.4245±0.1821	0.0698	0.91
S9C0.1304	5.0233±0.0590	0.1304	0.92
S10	0.0368±0.0053	0	1.45
S10C0.0361	2.5740±0.0647	0.0361	1.47
S10C0.0698	4.4531±0.1534	0.0698	1.54
S10C0.1304	5.1584±0.0557	0.1304	1.56
S11	0.0281±0.0027	0	1.52
S11C0.0698	4.2857±0.3074	0.0698	1.54
S12	0.1363±0.0176	0	1.46
S12C0.0216	3.2078±0.0354	0.0216	1.46
S12C0.0423	4.6075±0.0341	0.0423	1.47
S12C0.0811	4.6983±0.1203	0.0811	1.51
S12C0.15	4.9707±0.1562	0.15	1.55
S13	0.0121±0.0007	0	1.28
S13C	Limited by solubility	-	-

N/A- not applicable

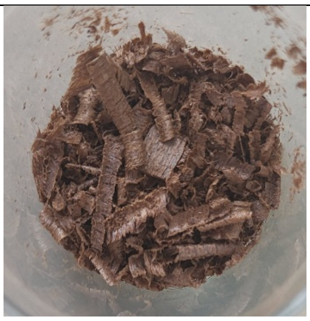

(a)

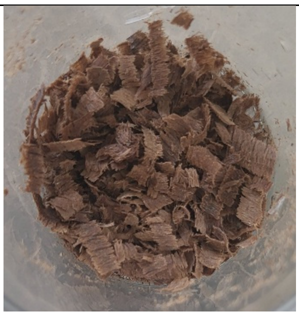

(b)

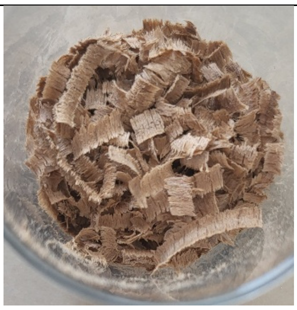

(c)

Figure S2. Visual appearance of WRT chips before and after extraction under different extraction conditions: (a) WRT chips before extraction; (b) WRT chips after 24 h of extraction in $S10C_{0.1304}$; and (c) WRT chips after 24 h of extraction in $S4C_{0.15}$.

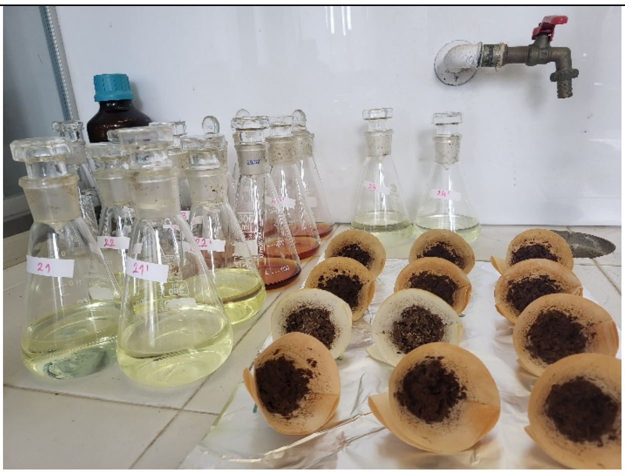

(a)

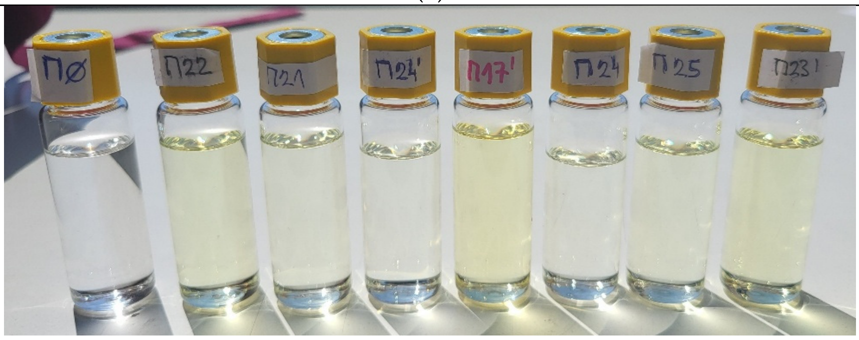

(b)

Figure S3. (a) WRT chips during the sample preparation and extraction process and (b) final extracts in *n*-hexane.
